# CHARMS and PROBAST at your fingertips: a template for data extraction and risk of bias assessment in systematic reviews of predictive models

**DOI:** 10.1186/s12874-023-01849-0

**Published:** 2023-02-17

**Authors:** Borja M. Fernandez-Felix, Jesus López-Alcalde, Marta Roqué, Alfonso Muriel, Javier Zamora

**Affiliations:** 1 Clinical Biostatistics Unit, Hospital Universitario Ramón y Cajal. IRYCIS, Madrid, Spain; 2 CIBER Epidemiology and Public Health (CIBERESP), Madrid, Spain; 3 Institute for Complementary and Integrative Medicine, University Hospital Zurich and University of Zurich, Zurich, Switzerland; 4 Faculty of Health Sciences, Universidad Francisco de Vitoria (UFV), Madrid, Spain; 5 Iberoamerican Cochrane Centre - Sant Pau Biomedical Research Institute (IIB-Sant Pau), Barcelona, Spain; 6 Department of Nursing and Physiotherapy, Universidad de Alcala de Henares, Alcala de Henares, Spain; 7 Institute of Metabolism and Systems Research, WHO Collaborating Centre for Global Women’s Health, University of Birmingham, Birmingham, UK

**Keywords:** CHARMS, PROBAST, Systematic review, Prognostic model, Template

## Abstract

**Background:**

Systematic reviews of studies of clinical prediction models are becoming increasingly abundant in the literature. Data extraction and risk of bias assessment are critical steps in any systematic review. CHARMS and PROBAST are the standard tools used for these steps in these reviews of clinical prediction models.

**Results:**

We developed an Excel template for data extraction and risk of bias assessment of clinical prediction models including both recommended tools. The template makes it easier for reviewers to extract data, to assess the risk of bias and applicability, and to produce results tables and figures ready for publication.

**Conclusion:**

We hope this template will simplify and standardize the process of conducting a systematic review of prediction models, and promote a better and more comprehensive reporting of these systematic reviews.

**Supplementary Information:**

The online version contains supplementary material available at 10.1186/s12874-023-01849-0.

## Background

Systematic reviews of clinical prediction model studies are becoming increasingly popular. Prediction models are covered by the type III prognostic research studies proposed by the PROGRESS (PROGnosis RESearch Strategy) partnership [1, 2]. The most common aims of these systematic reviews are to identify and summarize all available models for a particular target population, condition or outcome, and to summarize the predictive performance of a specific prognostic model while identifying potential sources of heterogeneity [3]. During the systematic review process, it is crucial for reviewers to extract key data from the relevant studies. Data extraction provides the reviewer the necessary information for describing and summarizing the findings, and examining the risk of bias and any applicability concerns of the models. Risk of bias refers to the likelihood that a primary predictive model study leads to a distorted, usually overly optimistic, estimate of predictive performance. Applicability concerns arise when a primary study question differs from the specific review question in terms of population, predictors or outcomes. Several checklists and toolkits have been developed to guide the process of data extraction and risk of bias assessment for different types of review questions [4].

The CHARMS checklist (CHecklist for critical Appraisal and data extraction for systematic Reviews of prediction Modelling Studies) provides guidance for both formulating the review question, and for extracting data the primary studies reporting prediction models [5].

The PROBAST tool (Prediction model Risk Of Bias Assessment Tool) is a checklist for assessing the risk of bias and the applicability of prognostic model studies [6, 7]. The PROBAST includes four domains: participants, predictors, outcome, and analysis. For each domain the tool provides signalling questions for determining whether the risk of bias and the applicability should be graded as low, high or unclear.

With the aim of facilitating the use of these two tools (i.e. CHARMS and PROBAST) for reviewers performing a systematic review of clinical prediction model studies, we have created an Excel template for extracting data and assessing the risk of bias and the applicability of predictive models.

## Implementation

The Excel file (named CHARMS and PROBAST template.xls) consists of eight sheets. The first sheet “*Home*” provides a description of the Excel file, instructions for its use and links to relevant papers and forms. The following three sheets (“*Summary*”, “*CHARMS*” and “*PROBAST*”) correspond to the collection of data from the studies included in the systematic review, and the following three sheets (“*Study Characteristics*”, “*Model characteristics* “, and “*PROBAST summary*”) contain the tables and figures generated from the data collected. The final sheet (“*CHARMS. Drop-down response lists*”) allows tailoring of the template to the systematic review. A more detailed description of each sheet is presented next.

To start with the data extraction process, for each predictive model presented in each study included in the systematic review, the user should tick the “new model” box on the “*Summary*” sheet. This operation enables the CHARMS and PROBAST forms for this new model in the corresponding sheets. The Excel template assumes that each study in the review reports a single prognostic model, but it can easily be generalized to a study reporting two or more models. In that case, the reviewer shall enable as many rows in the template as models are reported in that study. In the “*Summary*” sheet the following basic information of the new study should be filled in: author, year, title or an identifier (i.e. PMID or DOI), journal of publication and name of the model, if applicable. An identifier for each model is automatically created based on author name and year. In the last two columns of this summary sheet, the reviewer finds information on the status (i.e. complete or incomplete) of the CHARMS and PROBAST sheets.

The “*CHARMS*” sheet contains the template from Moons et al. [4]. The data extraction sheet is structured according to the eleven CHARMS domains: source of data, participants, outcome to be predicted, candidate predictors, sample size, missing data, model development, model performance, model evaluation, results and interpretation. To complete the data extraction process reviewers should fill in all the cells shaded in yellow. Depending on the item, the reviewers can choose from a drop-down list of options, or they can enter a free-text response. The items with available drop-down lists are showed in the last sheet of the Excel file (sheet named “CHARMS. *Drop-down response lists*”). The categories of these default lists can be tailored by the reviewer. When the information in the study report is not available, the reviewer has to fill in the cell with “No information”. In the participant description section, reviewers can specify the relevant characteristics that they plan to extract from the primary studies, tailored to the target population in the review. These characteristics will be the same for all models included in the review. For each domain within CHARMS, its status is incomplete whenever a cell within that domain remains empty (marked in yellow). In the observations section of the CHARMS checklist table (bottom part of CHARMS sheet), the reviewer will find a status line that flags each model as *“All information has been successfully registered”* when all domains are complete, or *“Incomplete data extraction”* otherwise. Additional information of the model could be extracted and filled in as free text on an additional information field at the bottom line. When all relevant information from a model has been extracted for all domains in the form, the CHARMS checklist for that model is flagged as complete in the “*Summary*” sheet.

The “*PROBAST*” sheet contains the template from Wolff et al. [6]. To make information of the model accessible to the reviewers, relevant information (such as source of data, inclusion and exclusion criteria, validation methods, performance measures, etc.) from CHARMS domains are automatically transferred into the “*PROBAST*” sheet. Reviewers should fill in signalling questions for all PROBAST domains: participants, predictors, outcome and analysis. These questions are shaded in yellow and responses should be selected from a drop-down list with the following categories “Yes”, “Probably yes”, “Probably no”, “No” or “No information”. Once all signalling questions for one domain have been filled, the risk of bias and applicability assessment cells become editable. Reviewers should rate risk of bias and concerns for applicability of the model as “Low”, “High” or “Unclear” for both. When the risk of bias assessment and the applicability of a model have been rated for all domains in the form, the PROBAST assessment for the model is flagged as complete in the “*Summary*” sheet.

## Results

In this section we present a worked example of the template file. This example is based on the data from a systematic review of prognostic models for mortality after cardiac surgery in patients with infective endocarditis [8].

Once we have extracted the data of the models included in the review using the corresponding CHARMS sheet (see Table 1 with data extracted from one of the models as an example) and after completion of the risk of bias assessment using PROBAST sheet (see Table 2 with the risk of bias assessment of the same model), the reviewers could obtain a number of tables and figures aimed to assist in the process of reporting adequately the review findings. All tables and figures can be copied and pasted for further editing.

**Table 1:**
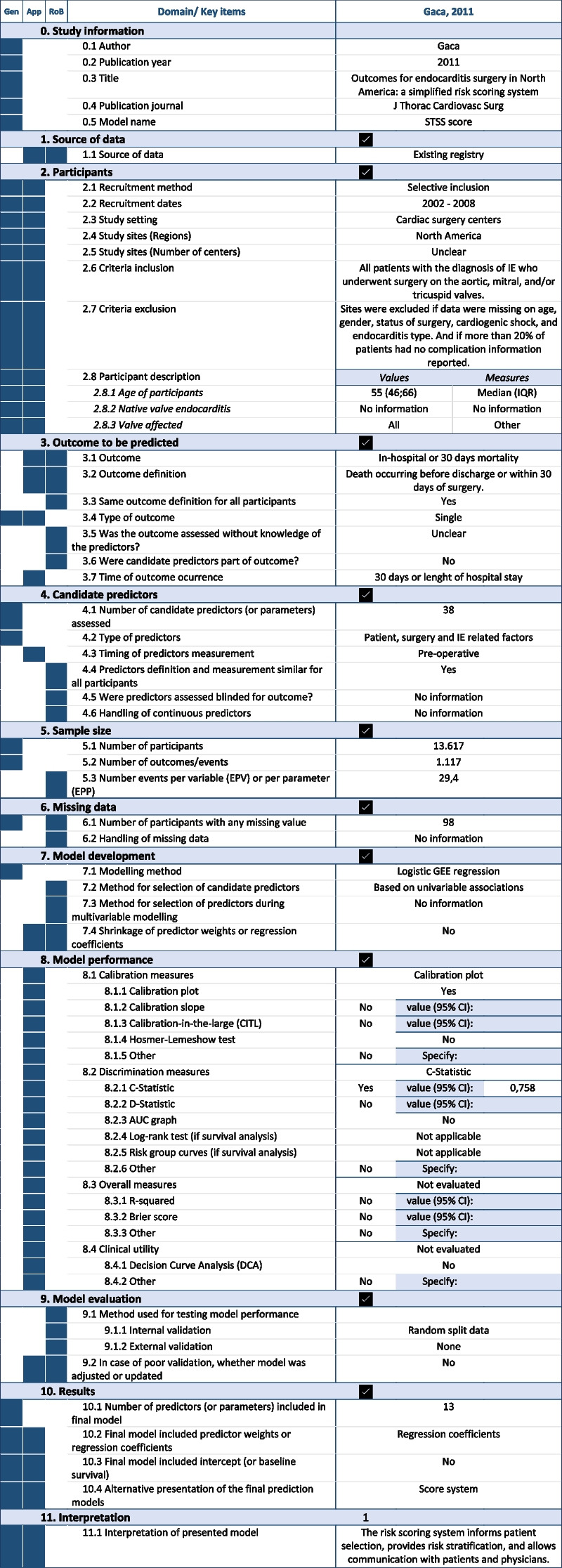
Example of CHARMS sheet using data from a primary study included in the systematic review of prognostic models for mortality after cardiac surgery in patients with infective endocarditis [8]

*Abbreviations*: *Gen* General description, *App* Applicability, *RoB* Risk of Bias

**Table 2:**
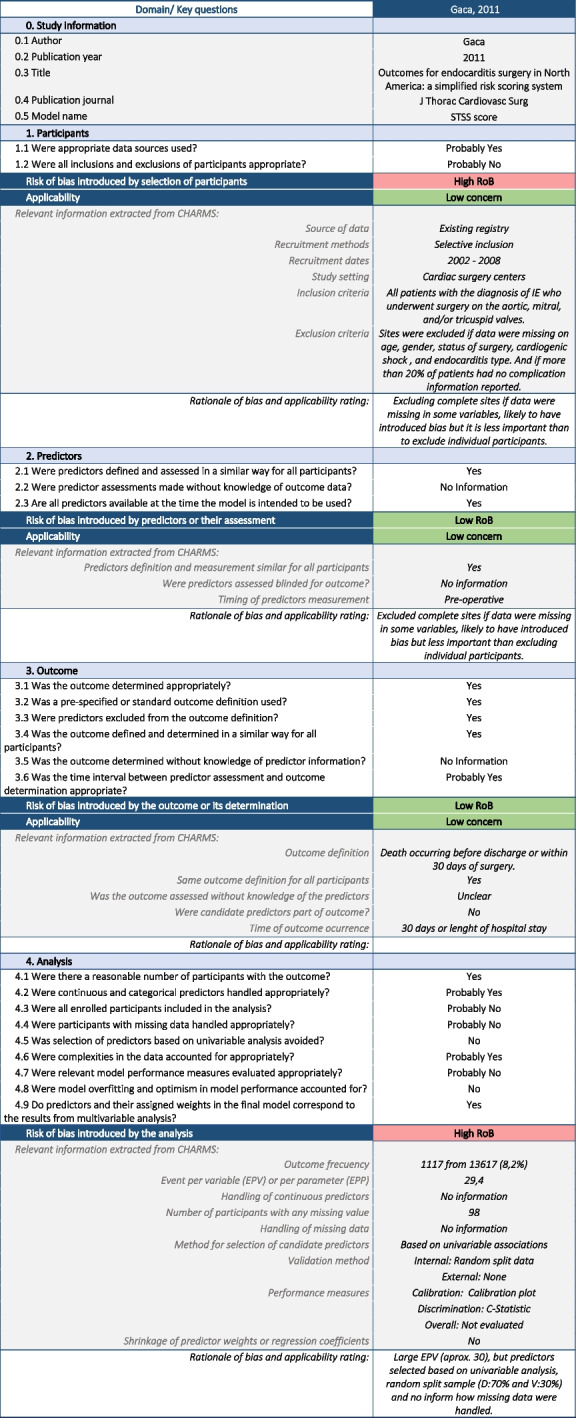
Example of PROBAST sheet using data from a primary study included in the systematic review of prognostic models for mortality after cardiac surgery in patients with infective endocarditis [8]

Gray shaded cells are automatically filled based on the information included in the CHARMS sheet

The first result table automatically created (sheet named: “*Study characteristics*”) shows a summary of the characteristics of included studies listed in the “*Summary*” sheet. It presents information covered by methods section (items 4 and 5) and results section (item 13) of the TRIPOD (Transparent Reporting of a multivariable prediction model for Individual Prognosis Or Diagnosis) statement [9]. The headers of the table include the source of data, the enrolment period, study setting and regions, and the participant characteristics previously pre-defined in the CHARMS sheet, in our example, these characteristics includes age, specification of native valve endocarditis and valves affected (see Table 3 with characteristics of the studies included in the review).

**Table 3:** Example of the table with study characteristics automatically produced by the Excel file using data from the systematic review of prognostic models for mortality after cardiac surgery in patients with infective endocarditis [8]

**Author, Year**	**Source of data**	**Enrolment period**	**Study setting**	**Study region**	Participant characteristics		
					**Age of participants**	**Native valve endocarditis**	**Valve affected**
Gaca, 2011 [10]	Existing registry	2002—2008	Cardiac surgery centers	North America	55 (46;66)	No information	All
De Feo, 2012 [11]	Retrospective cohort	1980—2009	Cardiac surgery center	Italy	49 (16)	440 (100)	All
Martínez-Sellés, 2014 [12]	Existing registry	2008—2010	Cardiac surgery centers	Spain	61.4 (15.5)	267 (61.1)	All
Madeira, 2016 [13]	Retrospective cohort	2007—2014	Cardiac surgery center	Portugal	60 (47;70)	94 (73.4)	All
Gatti (a), 2017 [14]	Other (specify)	2000—2015 (Italy) 2008 (France)	Cardiac surgery centers	Italy and France	59.1 (15.4)	285 (78.9)	All
Gatti (b), 2017 [14]	Other (specify)	2000—2015 (Italy) 2008 (France)	Cardiac surgery centers	Italy and France	59.1 (15.4)	285 (78.9)	All
Di Mauro, 2017 [15]	Retrospective cohort	2000—2015	Cardiac surgery centers	Italy	59.6 (15.1)	2.221 (82)	All
Gatti (c), 2017 [16]	Retrospective cohort	1999—2015	Cardiac surgery center	Italy	60.6 (8.5)	103 (74.6)	All
Olmos, 2017 [17]	Retrospective cohort	1996—2014	Cardiac surgery centers	Spain	62 (14)	259 (61.1)	Aortic / Mitral
Fernández-Hidalgo (a), 2018 [18]	Retrospective cohort	2000—2011	Cardiac surgery centers	Spain	58 (15.1)	No information	All
Fernández-Hidalgo (b), 2018 [18]	Retrospective cohort	2000—2011	Cardiac surgery centers	Spain	58 (15.1)	No information	All

The second table of results (sheet named: “*Model characteristics*”) shows the relevant information of the predictive models included in the review. It presents information about the methods section (items 7, 8 and 10) and the results section (item 14) of the TRIPOD statement. In addition, for each included model, a summary of the results of the risk of bias assessment and applicability is shown (see Table 4 with the characteristics of the models reviewed).

**Table 4:**
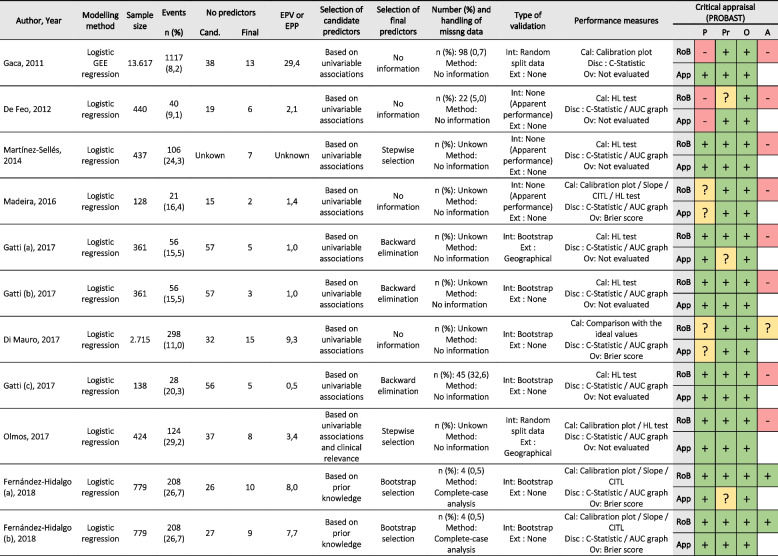
Example of the table with model characteristics automatically produced by the Excel file, using data from the systematic review of prognostic models for mortality after cardiac surgery in patients with infective endocarditis [8]

*Abbreviations: GEE* Generalized Estimating Equation, *n*: number of event and number of missing data, *Cand* Number of candidate predictors assessed, *EPV* Events per variable, *EPP* Events per parameter, Critical appraisal domains (*P* Participants, *Pr* Predictors, *O* Outcome, *A* Analysis), *Int* Internal validation, *Ext* External validation, *Disc* Discrimination, *Cal* Calibration, *Ov* Overall, *CITL* Calibration-in-the-large, *C*: C-Statistic, *AUC* Area under curve, *HL* Hosmer–Lemeshow, *RoB* Risk of Bias, *App* Applicability

+ Low RoB or low corcern for applicability

- High RoB or high concern for applicability

? Unclear RoB or applicability

**Fig. 1:**
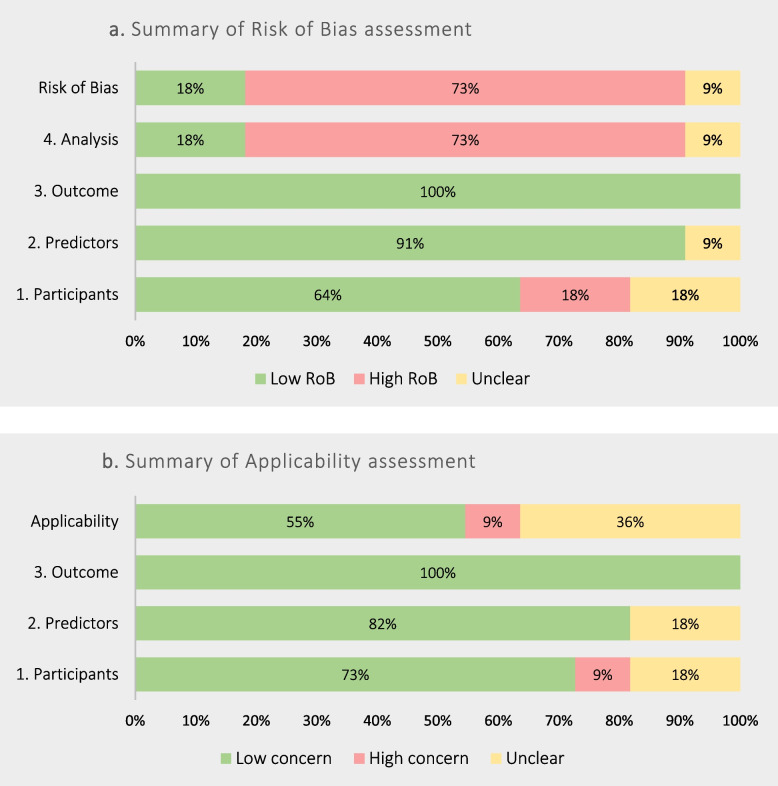
Example of the graph with the summary of PROBAST tool automatically produced by the Excel file using data from the systematic review of prognostic models for mortality after cardiac surgery in patients with infective endocarditis [8]

The sheet named "*PROBAST summary*” presents a table and a graph with the results of the risk of bias and applicability assessments (Table 5 and Fig. 1).

**Table 5:** Example of the table with the summary of PROBAST tool automatically produced by the Excel file using data from the systematic review of prognostic models for mortality after cardiac surgery in patients with infective endocarditis [8]

Author, Year	Risk of Bias				Applicability			Overall	
	1. Participants	2. Predictors	3. Outcome	4. Analysis	1. Participants	2. Predictors	3. Outcome	**Risk of Bias**	**Applicability**
Gaca, 2011 [10]	-	+	+	-	+	+	+	-	+
De Feo, 2012 [11]	-	?	+	-	-	+	+	-	-
Martínez-Sellés, 2014 [12]	+	+	+	-	+	+	+	-	+
Madeira, 2016 [13]	?	+	+	-	?	+	+	-	?
Gatti (a), 2017 [14]	+	+	+	-	+	?	+	-	?
Gatti (b), 2017 [14]	+	+	+	-	+	+	+	-	+
Di Mauro, 2017 [15]	?	+	+	?	?	+	+	?	?
Gatti (c), 2017 [16]	+	+	+	-	+	+	+	-	+
Olmos, 2017 [17]	+	+	+	-	+	+	+	-	+
Fernández-Hidalgo (a), 2018 [18]	+	+	+	+	+	?	+	+	?
Fernández-Hidalgo (b), 2018 [18]	+	+	+	+	+	+	+	+	+

The template as well as a filled in file with an example is provided as supplementary material, and this version and further updates can be downloaded from https://github.com/Fernandez-Felix/CHARMS-and-PROBAST-template.

## Discussion

We present in this manuscript an Excel template for extracting data and assessing the risk of bias and applicability of predictive modelling studies.

This template is the first to combine the CHARMS and PROBAST tools into one file. The template simplifies and standardizes the tasks of data extraction and risk of bias assessment, reducing the risk of errors and increasing reliability between data extractors. Having the relevant information at hand while assessing the risk of bias will make the review process more efficient. The template is easy to use and allows the reviewers to fill the forms using drop-down lists that are easily customisable. Such customisation makes our template versatile and adaptable to meet users' needs. The template generates several summary tables that can be used directly for publication with minor edits. All these characteristics will speed up the process of performing some of the steps of a systematic review and reporting its findings; surely, systematic reviewers will appreciate its usefulness.

There are some limitations to our template. First, it has been designed to include up to 30 existing models only (or 30 validation studies of a model). Second, the summary tables we produce are generic and might not fit every purpose. However, the tables could be edited outside the template to incorporate other aspects of interest for a specific review.

## Conclusion

We have designed a useful template for extracting data and assessing the risk of bias and the applicability of clinical prediction models using the CHARMS and PROBAST checklists. The template makes it easier for reviewers to manage these tools, and to produce results tables ready for publication with minor edits. We hope this template will promote a better and more comprehensive reporting of systematic reviews of prediction models. We encourage piloting the template and providing feedback to improve the template in future versions.

## Availability and requirements

Project name: None.

Project home page: None.

Operating system(s): Operating system with Microsoft Office.

Programming language: Only formulae available in Excel are employed.

Other requirements: None.

License: None required.

Any restrictions to use by non-academics: None.

## Supplementary Information

**Additional file 1.** CHARMS & PROBAST template.

**Additional file 2.** Example CHARMS and PROBAST template.

## Data Availability

All data generated or analysed during this study are included in this published article [and its supplementary information files]. CHARMS and PROBAST template.xls. Example CHARMS and PROBAST template.xls.

## Abbreviations

PROGRESS: PROGnosis RESearch Strategy
CHARMS: CHecklist for critical Appraisal and data extraction for systematic Reviews of prediction Modelling Studies
PROBAST: Prediction model Risk Of Bias Assessment Tool
TRIPOD: Transparent Reporting of a multivariable prediction model for Individual Prognosis Or Diagnosis

## References

[1] Croft P, Riley RD, van der Windt DA, Moons KG, Croft P, Riley RD, et al. 22A framework for prognosis research. In: Prognosis Research in Healthcare: Concepts, Methods, and Impact . Oxford University Press; 2019. 10.1093/med/9780198796619.003.0003

[2] Hemingway H, Croft P, Perel P, Hayden JA, Abrams K, Timmis A (2013). Prognosis research strategy (PROGRESS) 1: a framework for researching clinical outcomes. BMJ.

[3] van der Windt DA, Hemingway H, Croft P, Riley RD, Moons KG, Debray TP, et al. 208 Systematic reviews and meta-analysis of prognosis research studies. In: Prognosis Research in Healthcare: Concepts, Methods, and Impact. Oxford University Press; 2019. 10.1093/med/9780198796619.003.0010

[4] Roqué M, Martínez-García L, Solà I, Alonso-Coello P, Bonfill X, Zamora J (2020). Toolkit of methodological resources to conduct systematic reviews. F1000Research.

[5] Moons KGM, de Groot JAH, Bouwmeester W, Vergouwe Y, Mallett S, Altman DG (2014). Critical appraisal and data extraction for systematic reviews of prediction modelling studies: the CHARMS checklist. PLoS Med.

[6] Moons KGM, Wolff RF, Riley RD, Whiting PF, Westwood M, Collins GS (2019). PROBAST: a tool to assess risk of bias and applicability of prediction model studies: explanation and elaboration. Ann Intern Med.

[7] Wolff RF, Moons KGM, Riley RD, Whiting PF, Westwood M, Collins GS (2019). PROBAST: a tool to assess the risk of bias and applicability of prediction model studies. Ann Intern Med.

[8] Fernandez-Felix BM, Barca LV, Garcia-Esquinas E, Correa-Pérez A, Fernández-Hidalgo N, Muriel A (2021). Prognostic models for mortality after cardiac surgery in patients with infective endocarditis: a systematic review and aggregation of prediction models. Clin Microbiol Infect.

[9] Collins GS, Reitsma JB, Altman DG, Moons KGM (2015). Transparent Reporting of a multivariable prediction model for Individual Prognosis Or Diagnosis (TRIPOD). Ann Intern Med.

[10] Gaca JG, Sheng S, Daneshmand MA, O’Brien S, Rankin JS, Brennan JM (2011). Outcomes for endocarditis surgery in North America: a simplified risk scoring system. J Thor Cardiovasc Surg..

[11] De Feo M, Cotrufo M, Carozza A, De Santo LS, Amendolara F, Giordano S (2012). The need for a specific risk prediction system in native valve infective endocarditis surgery. Sci World J..

[12] Martínez-Sellés M, Muñoz P, Arnáiz A, Moreno M, Gálvez J, Rodríguez-Roda J (2014). Valve surgery in active infective endocarditis: a simple score to predict in-hospital prognosis. Int J Cardiol..

[13] Madeira S, Rodrigues R, Tralhão A, Santos M, Almeida C, Marques M (2016). Assessment of perioperative mortality risk in patients with infective endocarditis undergoing cardiac surgery: performance of the EuroSCORE I and II logistic models. Interact CardioVasc Thorac Surg..

[14] Gatti G, Perrotti A, Obadia J, Duval X, Iung B, Alla F, et al. Simple scoring system to predict in-hospital mortality after surgery for infective endocarditis. JAHA. 2017;6(7):e004806. 10.1161/JAHA.116.004806 PMC5586260 28729412

[15] Di Mauro M, Dato GMA, Barili F, Gelsomino S, Santè P, Corte AD (2017). A predictive model for early mortality after surgical treatment of heart valve or prosthesis infective endocarditis. The EndoSCORE. Int J Cardiol..

[16] Gatti G, Benussi B, Gripshi F, Della Mattia A, Proclemer A, Cannatà A (2017). A risk factor analysis for in-hospital mortality after surgery for infective endocarditis and a proposal of a new predictive scoring system. Infection..

[17] Olmos C, Vilacosta I, Habib G, Maroto L, Fernández C, López J (2017). Risk score for cardiac surgery in active left-sided infective endocarditis. Heart..

[18] Fernández-Hidalgo N, Ferreria-González I, Marsal JR, Ribera A, Aznar ML, de Alarcón A, et al. A pragmatic approach for mortality prediction after surgery in infective endocarditis: optimizing and refining EuroSCORE. Clin Microbiol Infect. 2018;24:1102.e7–15. 10.1016/j.cmi.2018.01.019 29408350

